# Development and clinical validation of a clinically translatable non-chip-on-tip transvaginal imaging system (GynoSight v2.0) for early detection of premalignant cervical lesions

**DOI:** 10.1117/1.JBO.30.10.106002

**Published:** 2025-10-27

**Authors:** Karthika Jeyachandran, Mohammed Ansar Pakirithodiyil, Keerthana Aruldoss, Milind Lal, Dhanush Koodi M, Arpitha Anantharaju, YuanYuan Sun, Rongguang Liang, Uttam M. Pal

**Affiliations:** a Indian Institute of Information Technology, Design and Manufacturing, Kancheepuram, Department of Sciences and Humanities, Chennai, Tamil Nadu, India; b Indian Institute of Information Technology, Design and Manufacturing, Kancheepuram, Department of Electronics and Communications, Chennai, Tamil Nadu, India; c National Institute of Engineering, Department of Mechanical Engineering, Mysore, Karnataka, India; d Jawaharlal Institute of Postgraduate Medical Education and Research, Department of Gynecology and Obstetrics, Puducherry, India; e The University of Arizona, Wyant College of Optical Sciences, Tucson, Arizona, United States

**Keywords:** multispectral imaging, non-chip-on-tip, transvaginal imaging probe, oxygen saturation, image quality quantification, image registration

## Abstract

**Significance:**

Cervical cancer is the fourth most common cancer among women globally. Hence, it is crucial to develop a noninvasive and portable optical imaging modality for the early detection of premalignant cervical lesions.

**Aim:**

We present the development and clinical validation of GynoSight v2.0, an indigenously developed multispectral, non-chip-on-tip source, hand-held, portable transvaginal imaging probe, for evaluating tissue health and identifying anomalies, such as those linked to precancerous cervical lesions.

**Approach:**

GynoSight v2.0 houses a 16 LEDs, 5-megapixel camera, and a Raspberry Pi 5 module. A comparative shadowing effect analysis was performed between GynoSight v2.0 and colposcopy by evaluating statistical metrics such as mean pixel intensity (MPI), shadow area percentage (SAP), entropy, and contrast-to-noise ratio. In addition, the relative oxygen saturation maps of the cervical tissue were computed from the multispectral registered image using the proposed discrete Fourier transform-based image registration technique.

**Results:**

The images of N=6 (N=2 normal, N=1 premalignant, and N=3 high-grade squamous intraepithelial lesion) subjects were acquired. A comparative shadowing analysis shows a 50-unit gray level value separation between the colposcope and GynoSight v2.0 images. The pixel values of the colposcope are skewed to the lower pixel values, and the pixel values of GynoSight v2.0 are spread uniformly over 0 to 255 gray-level pixel values. In addition, the statistical analysis showed that MPI, SAP, and entropy are significant metrics for shadowing effect quantification.

**Conclusions:**

The colposcopy images showed more shadowing effects than the GynoSight v2.0 images and hence provide better illumination to aid in better diagnosis.

## 1 Introduction

Cervical cancer is one of the most common cancers in the female population, especially in middle- and low-income countries where the regular screening of the cervix is limited and has limited diagnostic infrastructure.^1^ Studies have shown that cervical cancer affects the female population majorly in the age group of 15 to 44 years.^2^ The precancerous changes in the cervical cells are introduced with the persistent infection with the high-risk human papillomavirus (HPV). Cervical cancer progresses through various stages of cervical intraepithelial neoplasia (CIN), which involves CIN 1 (mild dysplasia), CIN 2 (moderate dysplasia), and CIN 3 (severe dysplasia/carcinoma *in situ*).^3^ Cervical cancer is preventable and manageable if it is diagnosed in the early stage. In addition, early detection reduces the chance of metastasis, which is when cancer spreads to other organs and becomes more challenging to treat. There are a few conventional diagnostic methods available for cervical cancer diagnosis, which include a Pap smear test, visual inspection of the cervix by the application of acetic acid and Lugol’s Iodine using colposcopy, HPV testing, and biopsy. These diagnostic techniques are subjective and rely on experienced personnel.

Thus, developments in optical imaging technologies, such as Raman spectroscopy, diffuse reflectance imaging, fluorescence imaging, optical coherence tomography, multispectral imaging, and hyperspectral imaging, particularly in environments with limited resources, create new opportunities for noninvasive, real-time, accurate, and reliable cervical cancer diagnosis.^4^^,^^5^ Balas^6^ demonstrated the feasibility of using multispectral imaging for cancer and precancerous cervical lesion detection. For instance, Asiedu et al.^7^^,^^8^ developed a novel speculum-free transvaginal imaging probe to visualize the cervix. This reduces the discomfort associated with traditional speculum-based cervical examination as it deters women from undergoing regular screening. This development is a component of a more significant cervical screening trend that uses patient-friendly technologies to improve patient comfort and accessibility, which could revolutionize standard screening procedures without compromising image quality. Complementing the design of a speculum-free transvaginal imaging probe, Asiedu et al.^9^ proposed an algorithm for automated detection of pre-cervical cancer lesions. Similarly, Boonya-ananta et al.^10^ designed a speculum-free polarized imaging system of the uterine cervix during pregnancy. Shukla et al.^11^^,^^12^ conducted a study that utilized fluorescence spectroscopy technology integrated with portable smartphone technology to classify different grades of cervical cancer.

Access to cervical cancer screening with standard colposcopy is still a significant problem in environments with inadequate resources. Recent advancements aim to overcome this by creating portable, reasonably priced gadgets that yield desired outcomes in resource-constrained environments. The Point of Care Tampon (POCkeT) Colposcope^13^ is one such invention that offers a cervical cancer screening platform with minimal infrastructure. The integration of colposcopy and high-resolution microendoscopy (HRME)^14^ enabled spatially aligned widefield and microscopic video acquisition for lesion detection and cellular analysis.

One of the key characteristics of the cancerous transformation of cervical tissue is increased vascularity. The increased vascularity arises from the tumor’s demand for nutrients and oxygen as it proliferates rapidly. Hence, it plays a pivotal role in the cancerous transformation.^15^^,^^16^ Recent advancements in optical techniques such as multispectral imaging^17^^–^^19^ and hyperspectral imaging^20^^,^^21^ help to quantify RBC tissue fraction. Such optical methods allow for noninvasive, real-time blood supply, and oxygenation visualization, offering a deeper understanding of cellular and vascular changes associated with early cancerous transformations. These innovations are especially valuable for identifying angiogenic patterns supporting early cancer detection.

This work presents a portable, cost-effective, multispectral, non-chip-on-chip light source, transvaginal imaging probe, an extension of our previous work by Karthika et al.^22^ This improved standalone imaging system integrates a 5-megapixel camera to capture cervix images at better resolution and a Raspberry Pi-5 module for capturing, processing, and displaying images on the monitor. The user interacts with the system through the graphical user interface (GUI) to facilitate user interaction and improve usability. The ratio metric analysis is used to get a relative oxygen saturation (OS) map of the cervix, which is a key biomarker for the assessment of early diagnosis of cervical cancer. In addition, a comparative analysis of the shadowing effect of GynoSight v2.0 and colposcopy images using various statistical metrics such as mean pixel intensity (MPI), shadow area percentage (SAP), entropy, and contrast-to-noise ratio (CNR) was discussed.

## 2 Materials and Methods

This section outlines the design of the transvaginal imaging system, image acquisition protocol, metrics of shadowing effect quantification, image registration algorithm, and relative OS map. A GUI was developed to acquire images from the patients and is described in Sec. S1 in the Supplementary Material. The imaging protocol used for acquiring the images from the patients has been described in Sec. S2 in the Supplementary Material.

### 2.1 Optical Probe Design

**Fig. 1:**
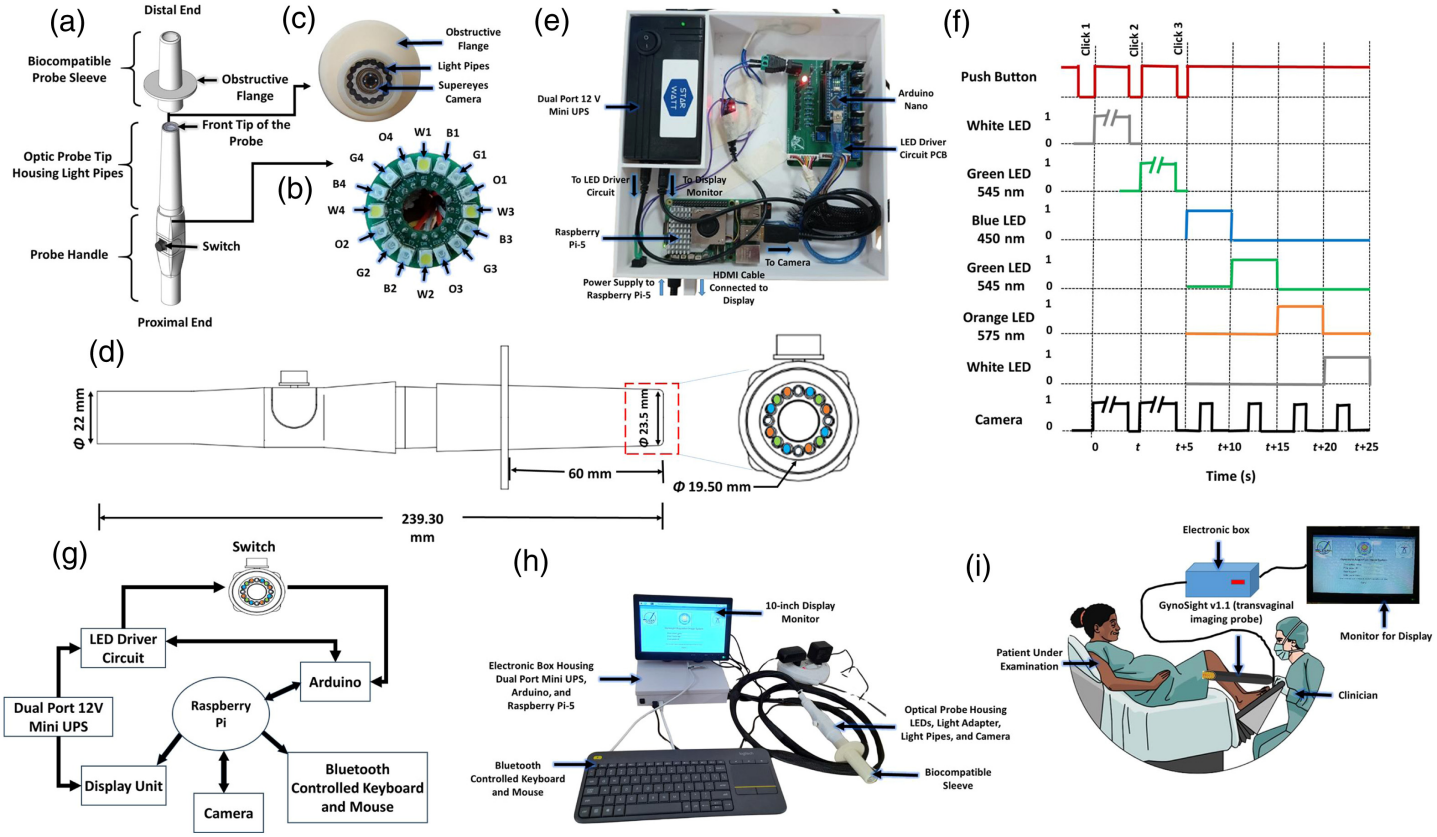
(a) Blown-up diagram of GynoSight v2.0 transvaginal imaging probe. (b) LED orientation in the probe, where the four LEDs are used for one wavelength of light. W1, W2, W3, and W4 for white light; B1, B2, B3, and B4 for blue light; G1, G2, G3, and G4 for green light; and O1, O2, O3, and O4 for orange light. (c) Front end of the probe. (d) GynoSight v2.0 probe design with annotated dimensions. (e) Electronic box of GynoSight v2.0. (f) Timing diagram of turning ON and OFF of LEDs and the camera. (g) Schematic representation of the prototype setup. (h) Hardware arrangement of GynoSight v2.0. (i) Schematic representation of image acquisition using GynoSight v2.0.

The optical probe has three parts, as shown in Fig. 1(a): (1) optical probe tip, (2) biocompatible probe sleeve, and (3) probe handle. The design of the non-chip-on-tip technology where the light source such as LEDs are placed in proximal end (outside the patient body) of the optic probe tip and light pipes are coupled to the LED with the coupling efficiency of the light pipe was reported to be more than 25% to transmit the light, helping in managing the overall temperature of the distal end (inside the patient body) optic probe tip.

The optical probe tip of 11.5 cm length houses the camera module (φ=9  mm) accommodated in the central aperture of 10 mm, and 16 light pipes (φ=2  mm) are accommodated in an annular configuration with an aperture of 2.8 mm. The biocompatible probe sleeve was fabricated using biocompatible material (MED-WHT 10) to eliminate the contamination of the probe tip. The sleeve has an obstructive flange placed 60 mm from the distant (front) end of the sleeve with a 3 mm thickness and a 6 cm diameter. The obstructive flange in Fig. 1(c) prevents the distal end of the optical probe from accidentally touching the surface of the cervix. The probe handle of 12.5 cm provides a broad foundation for an ergonomic grip. The probe accommodates an LED array, a coupling adapter, and a tactile switch. The transvaginal probe with the dimensions is shown in Fig. 1(d).

### 2.2 LED Array Configuration

The LED array configuration consists of LEDs operating in four wavelengths: white light (W), blue 450 nm (B), green 545 nm (G), and orange 610 nm (O). The LEDs of the same wavelength (e.g., white depicted by W1) are arranged in a quadrature configuration in an annular formation with an angular separation of 90 deg (W1, W2, W3, and W4), as shown in Fig. 1(b). This leads to a total of 16 LEDs housed inside the optic probe.

### 2.3 Camera and LED Driver Unit

The LED driver circuit is designed to facilitate the operation of multispectral LEDs with controlled light intensity using potentiometers and LM317 for delivering specific current to LEDs for a uniform illumination ellipse, capturing and displaying multispectral images by connecting the LED driver circuit, Arduino Nano, Raspberry Pi-5, and 10.1-in. display, as shown in Fig. 1(e). More details on the electric circuit are provided in our previous work.^22^

Figure 1(g) shows the functional block diagram; the system uses a dual-port 12V mini-UPS to power up the Raspberry Pi-5 and LED driver circuit. Raspberry Pi-5 module is connected to peripherals such as a Bluetooth-controlled keyboard and mouse, monitor, and camera. The Raspberry Pi-5 is also connected to Arduino and establishes two-way communication. The switch is connected to Arduino Nano, which enables switching ON/OFF of LEDs. The Raspberry Pi and Arduino Nano were both used because of the need to switch LEDs and acquire video and images through the camera simultaneously. The complete setup of the transvaginal imaging probe GynoSight v2.0 is shown in Fig. 1(h), whereas the tabletop setup in the clinical setup is shown in Fig. 1(i).

Table SI in the Supplementary Material gives detailed technical specifications for GynoSight v2.0.

### 2.4 Shadowing Effect Quantification Metrics

The acquired images from the Colposcope (Leisegang Feinmechanik Optik GmbH, Model 3MV) and GynoSight v2.0 were analyzed for shadowing effect using metrics such as MPI, SAP, entropy, and CNR.

The MPI was computed as the average intensity value of all pixels in the image. The higher and lower values of MPI indicate better illumination and shadowing area, respectively. The MPI of an image is calculated as follows:

$$\mathrm{MPI}=\frac{1}{N}\overset{N}{\underset{i=1}{\sum }}P_{i},$$ (1)

where $P_{i}=P_{1},P_{2},P_{3},\ldots ,P_{N}$ represents the pixel intensity values of an image, and N represents the total number of pixels in an image. The SAP was calculated to quantify the proportion of the image obscured by the shadow using binary thresholding, where the binary threshold value is 60 (0- to 60-pixel intensity levels representing low pixel intensity levels). Hence, this is defined as

$$\mathrm{SAP}=(\frac{\text{Total Pixels Below threshold}}{\text{Total Number of Pixels}})\;\times 100.$$ (2)

The entropy of an image was calculated to understand the randomness in an image. It is calculated using the equation below

$$\text{Entropy}=\overset{K}{\underset{l=1}{\sum }}P(l)\log_{2}\;P(l).$$ (3)

P(l) is the probability of the l’th intensity level, and K is the total number of intensity levels. Finally, CNR was computed to evaluate the ability to distinguish among different regions, i.e., the contrast between the region of interest (ROI) and background, given by

$$\mathrm{CNR}=\frac{\left|I_{\mathrm{ROI}}-I_{\text{background}}\right|}{I_{\text{background}}}.$$ (4)

$I_{\mathrm{ROI}}$ and $I_{\text{background}}$ are the mean intensities of ROI and the background of an image.

### 2.5 Image Registration

**Fig. 2:**
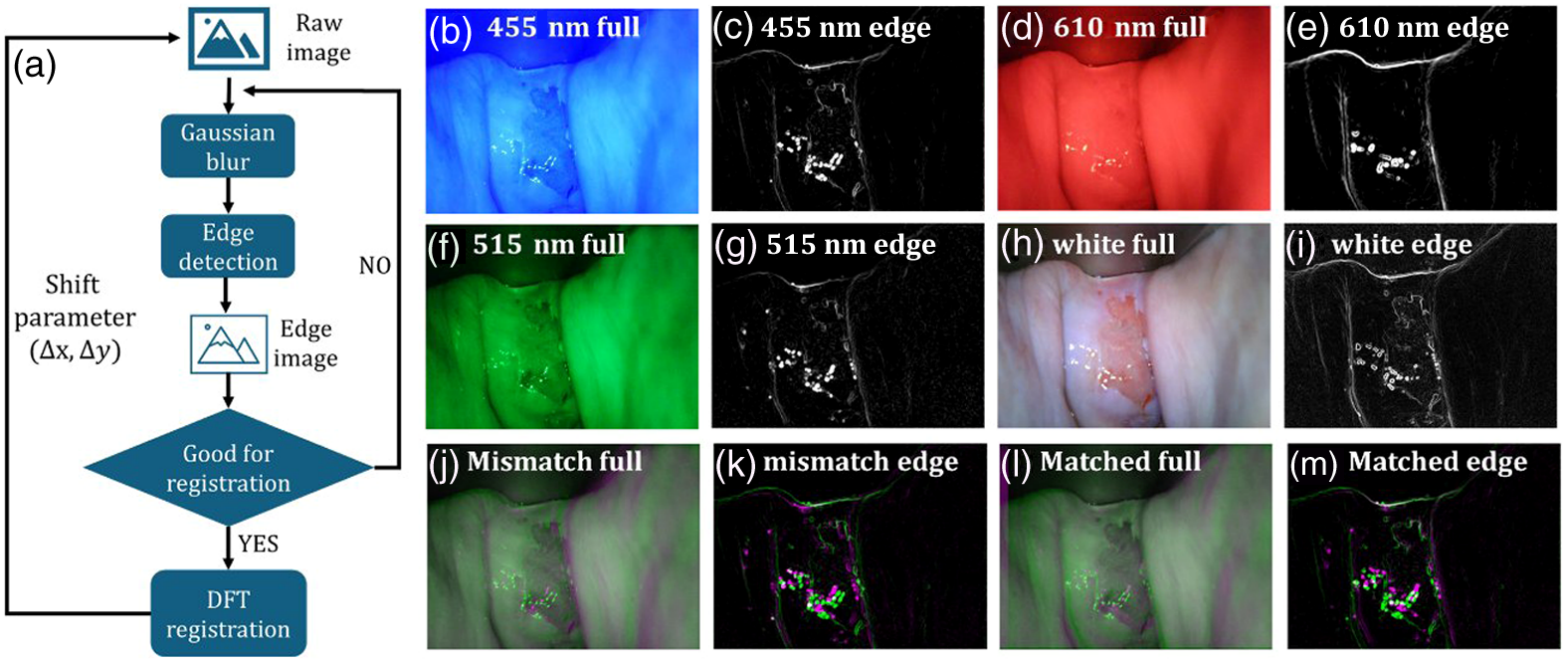
(a) Multispectral cervical image registration pipeline. (b), (d), (f), (h) The raw images were taken at 455, 515, and 610 nm and with a white LED. (c), (e), (g), (j) The produced edge images at 455, 515, and 610 nm and with white LED. (j)–(k) Color overlay full and edge images in a mismatch case. (l)–(m) Color overlay full and edge images in a good match case.

An image registration process is needed to obtain pixel-wise matched multispectral images before implementing the spatial map of RBC and the OS map. As the raw images were taken by switching LED sources time sequentially from the handheld device, there were unavoidable small spatial offsets between adjacent frames in the ROI because of hand motion, illumination angle changes, etc. However, most feature-based image registration methods fail for the obtained data due to local similarities and a lack of globally stable features in cervical images, making it difficult for the registration algorithms to find correct matches. To solve this problem, a unique registration pipeline for cervical multispectral images is proposed, as shown in Fig. 2. The raw image is blurred using a Gaussian kernel and fed into an edge detection algorithm. After edge detection, pixels of smooth tissues are removed, and global edges and unique shapes are emphasized for the subsequent correlation-based discrete Fourier transform algorithm.^23^ It is to be noted that the speckles appearing in the images were ignored in the registration because they vary due to factors including illumination and imaging angle. The algorithm calculates the offset in horizontal and vertical orientations according to the reference, and the shift parameters are applied to the original full image. If one cycle of Gaussian blurring and edge detection cannot produce a clear edge image, the intermediate edge image is fed into a new cycle again, except that a smaller Gaussian kernel is utilized. The reason for first applying a Gaussian blur before edge detection is that the obtained image suffers from a low signal-to-noise ratio. Without Gaussian blur preprocessing, edge detection would amplify noise pixels, interfering with subsequent registration.

### 2.6 Oxygen Saturation Map

OS maps provide critical information about tissue oxygenation levels. Deviations from normal values can indicate underlying health issues, such as ischemia, hypoxia, or hyperoxia. A relatively normalized OS map can be calculated for analysis with the multispectral cervical images using the two-wavelength oximetry method.^24^ With the obtained spectral images at three wavebands, the OS map can be estimated based on the absorption difference between oxygenated hemoglobin (${\mathrm{HbO}}_{2}$) and deoxygenated hemoglobin (Hb), with the mathematical definition as

$$O=\frac{C_{{\mathrm{HbO}}_{2}}}{C_{{\mathrm{HbO}}_{2}}+C_{\mathrm{Hb}}}.$$ (5)

The method of two-wavelength-based oximetry is applied, which is based on the Beer–Lambert law of light propagation through a medium

$$I_{\lambda }=I_{\lambda_{0}}\;\exp(-c\epsilon_{\lambda }d),$$ (6)

where $I_{\lambda }$ and $I_{\lambda_{0}}$ represent the reflected and incident light at wavelength λ; c is the molar concentration of hemoglobin within the blood, $\epsilon_{\lambda }$ is the wavelength-dependent molar extinction coefficient of absorbers, and d denotes the optical path length. The main absorbers in our model consist of ${\mathrm{HbO}}_{2}$ and Hb, with the format of

$$\epsilon_{\lambda }=\frac{C_{{\mathrm{HbO}}_{2}}}{C_{{\mathrm{HbO}}_{2}}+C_{Hb}}\cdot \epsilon_{\lambda {\mathrm{HbO}}_{2}}+\frac{C_{Hb}}{C_{{\mathrm{HbO}}_{2}}+C_{Hb}}\cdot \epsilon_{\lambda Hb}\;=SO\cdot \epsilon_{{\mathrm{HbO}}_{2}}+(1-SO)\cdot \epsilon_{\lambda Hb},$$ (7)

where $\epsilon_{{\mathrm{HbO}}_{2}}$ and $\epsilon_{\lambda Hb}$ are the molar extinction coefficients of fully oxygenated hemoglobin and deoxygenated hemoglobin, respectively, taking the logarithm of both sides of Eq. (6) yields the optical density (OD) at a specific wavelength.

$$OD_{\lambda }=-\log(\frac{I_{\lambda }}{I_{\lambda_{0}}})=cd\epsilon_{\lambda }.$$ (8)

The final OS map is linearly proportional to the OD ratio (ODR), which is defined as

$$OS\propto ODR=\frac{OD_{\lambda_{1}}}{OD_{\lambda_{1}}}=\frac{cd\epsilon_{\lambda_{1}}}{cd\epsilon_{\lambda_{2}}}=\frac{\epsilon_{\lambda_{1}}}{\epsilon_{\lambda_{2}}}.$$ (9)

Given that both c and d are independent of wavelength in our model. In this study, the ODR is taken between OD at 450 nm and OD at 610 nm.

Given the assumption of negligible scattering in the current two-wavelength oximetry model,^24^ proven by relevant works,^25^^–^^28^ the resulting OS maps are best interpreted as a relative OS map that reflects spatial variations rather than exact physiological values.

## 3 Results and Discussion

**Fig. 3:**
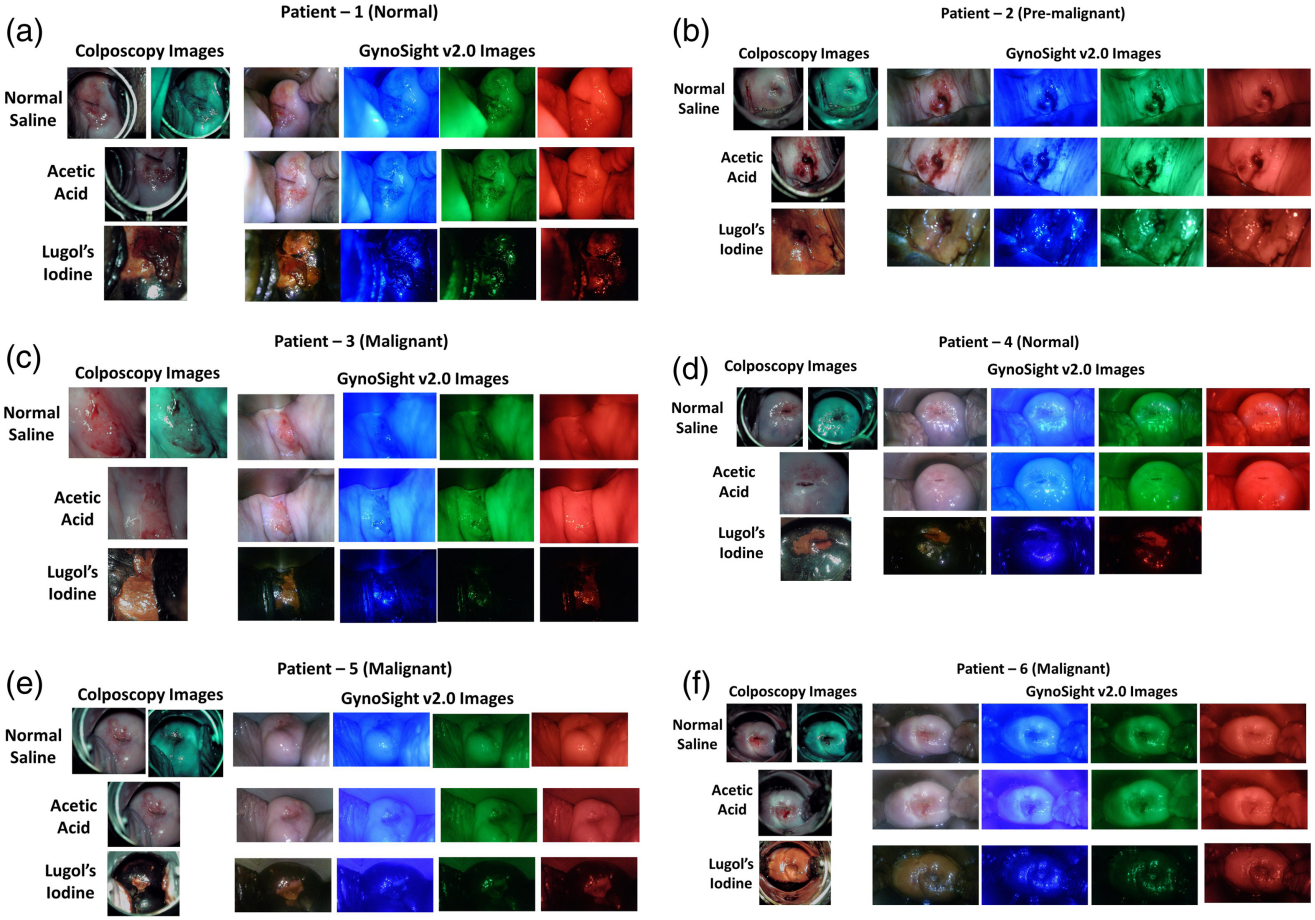
Colposcopy images (left)- normal saline images, acetic acid, and lugol’s iodine images, and multispectral images (right) using white light, 450, 545, and 610 nm for normal saline, acetic acid, and lugol’s iodine of (a) patient 1: This is a normal subject. (b) Patient 2: This is a subject diagnosed as premalignant. (c) Patient 3: This is a malignant subject. (d) Patient 4: This is a normal subject. (e) Patient 5: This is a malignant subject. (f) Patient 6: This is a malignant subject.

The study was performed at JIPMER, Puducherry, India, with informed written consent from human participants with an Institutional Ethical Clearance certificate number JIP/IEC/2023/04/043. The study was performed on N=6 human participants (N=2 normal, N=1 premalignant, and N=3 malignant). The colposcopy and GynoSight v2.0 images of patients 1 (normal), patient 2 (premalignant), patient 3 (malignant), patient 4 (normal), patient 5 (malignant), and patient 6 (malignant) are shown in Figs. 3(a)–3(f), respectively.

This section focuses on two primary analyses: quantification of the shadowing effect and statistical analysis for the shadowing effect of images from GynoSight v2.0 and colposcopy.

### 3.1 Quantification of Shadowing Effect

**Fig. 4:**
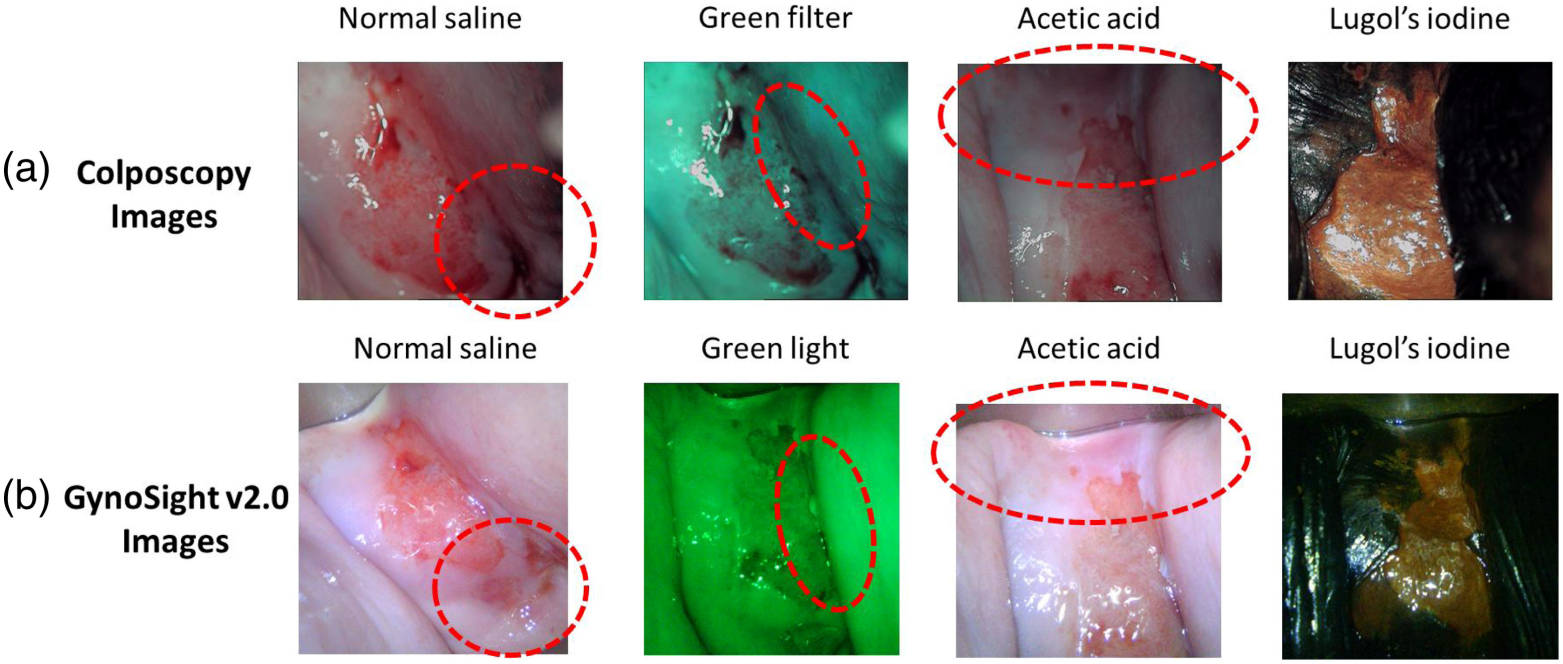
Patient 3: (a) Colposcopy white light images of the cervix after application of normal saline, green filter, acetic acid, and lugol’s iodine. (b) GynoSight v2.0 white light images of the cervix after application of normal saline, green light, acetic acid, and lugol’s iodine. The red circular dashed lines show the region of lower illumination of Colposcopy and the region of improved illumination in GynoSight v2.0 images, highlighting the reduced shadowing effect.

The shadowing effect in imaging occurs when certain areas of an image appear darker due to partial or complete light obstruction. In cervix imaging, especially colposcopy procedures, shadowing can obscure essential details, reducing the visibility of critical regions. As shown in Fig. 4, the colposcopy images of patient 3 exhibit areas of poor illumination, especially under normal saline, green filter, and acetic acid, where parts of the cervix fall into shadow (highlighted by red dashed circles). By contrast, Fig. 4(b) shows the GynoSight v2.0 images of patient 3, which show improved illumination in the same region. The shadow region contains less information than the nonshadow region. For accurate diagnosis, addressing the shadowing effect is crucial to ensure high-quality images for effective diagnosis. In this work, the shadowing effect is quantified and compared between Colposcope and GynoSight v2.0, objectively evaluating the impact of shadow on the image quality.

**Fig. 5:**
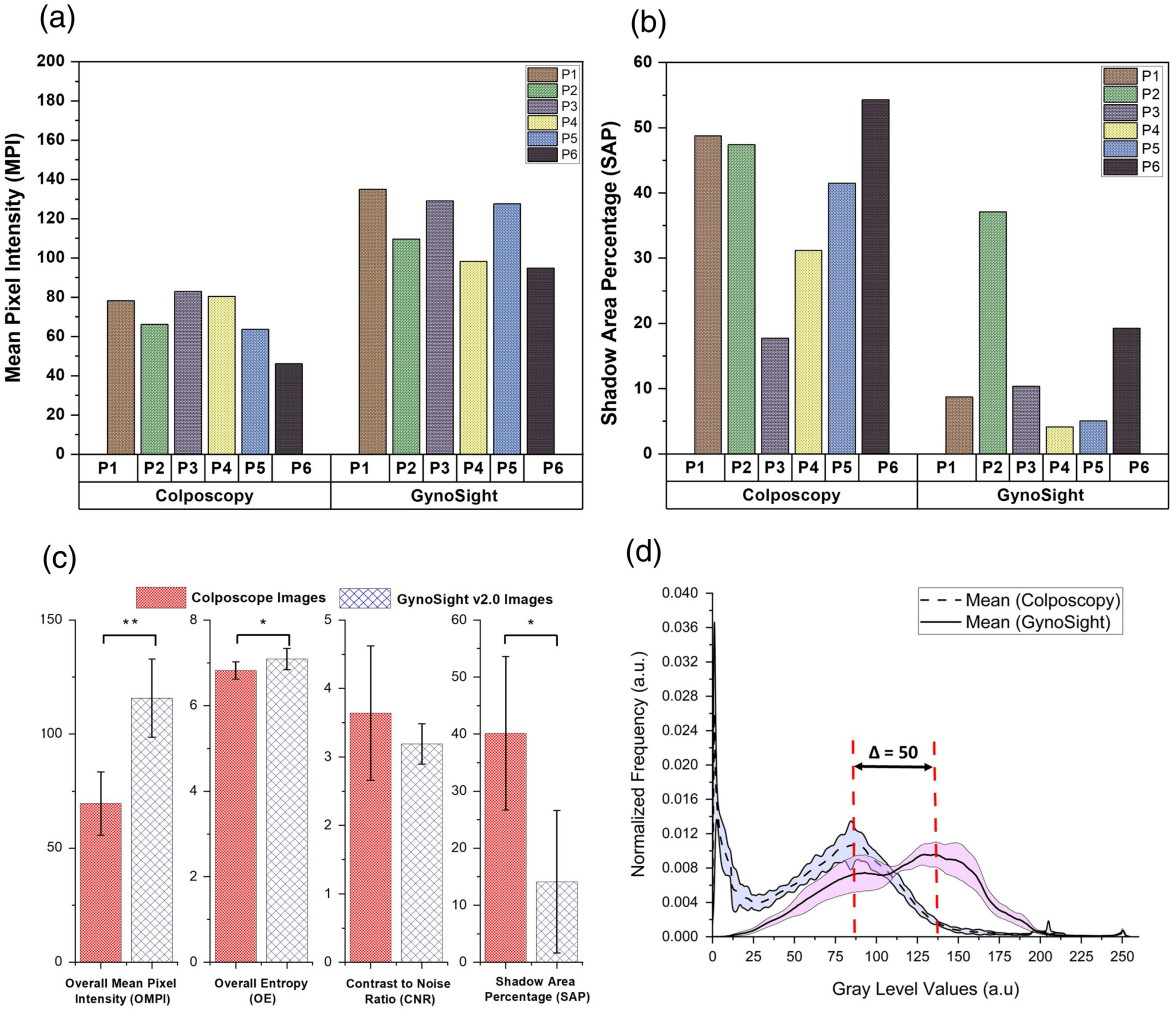
Statistical analysis of the shadowing effect. Bar chart representation of (a) MPI, (b) SAP, (c) overall mean pixel intensity (OMPI), overall shadow area percentage (OSAP), overall entropy (OE), and overall contrast-to-noise ratio of colposcopy and GynoSight v2.0 images. The asterisk shows the level of significance. (d) Normalized frequency versus gray level values of colposcopy and GynoSight v2.0 images of the cervix for all six patients.

The shadowing effect was quantified through normalized frequency versus gray level values analysis of the acquired colposcopy and GynoSight v2.0 images, as shown in Fig. 5(d). For the colposcopy images, the pixel distribution is shifted toward the left-hand side of the graph, i.e., lower pixel intensity values, indicating lower intensity values due to nonuniform illumination caused by shadowing; conversely, the images obtained using GynoSight v2.0 exhibit a shift of pixel distribution toward the right-hand side. The peak-to-peak separation of gray level values between the colposcope and GynoSight v2.0 was reported to be significant (50 units).

The MPI, standard deviation, entropy, CNR, and SAP were estimated to evaluate image quality, quantify shadowing effects, and guide improvements in image acquisition, as shown in Table SII in the Supplementary Material. A Mann–Whitney U-test was performed to determine the statistical significance of the individual metrics. The p-values were 0.002, 0.041, 0.699, and 0.015 for MPI, entropy, CNR, and SAP, respectively. The results suggest that MPI, entropy, and SAP are statistically significant metrics for distinguishing between the groups, whereas CNR does not show a significant difference. Figure 5(a) illustrates the comparison of the MPI of images from colposcopy and GynoSight v2.0 of six subjects using an error bar graph, and Fig. 5(c) illustrates the overall mean pixel intensity (OMPI) across six patients of colposcope and GynoSight v2.0 images. The graph shows that GynoSight v2.0 has the highest OMPI of 115.75 compared with colposcope images with an OMPI of 69.58. These findings suggest that most of the pixel intensity of GynoSight v2.0 images has higher gray values, in other words, yielding a brighter image for visualization of inaccessible regions. In addition, Fig. 5(b) compares the SAP of six patients’ images from the colposcope and GynoSight v2.0. By contrast, Fig. 5(c) compares the overall shadow area percentage (OSAP) of images from colposcope and GynoSight v2.0 for six patients. The results show that the OSAP of the colposcope exhibits the highest value of 40.15, and GynoSight v2.0 exhibits the lowest value of 14.11. This indicates that GynoSight v2.0 has better illumination and minimized obscured regions.

### 3.2 Analysis of the Relative Oxygen Saturation Map

**Fig. 6:**
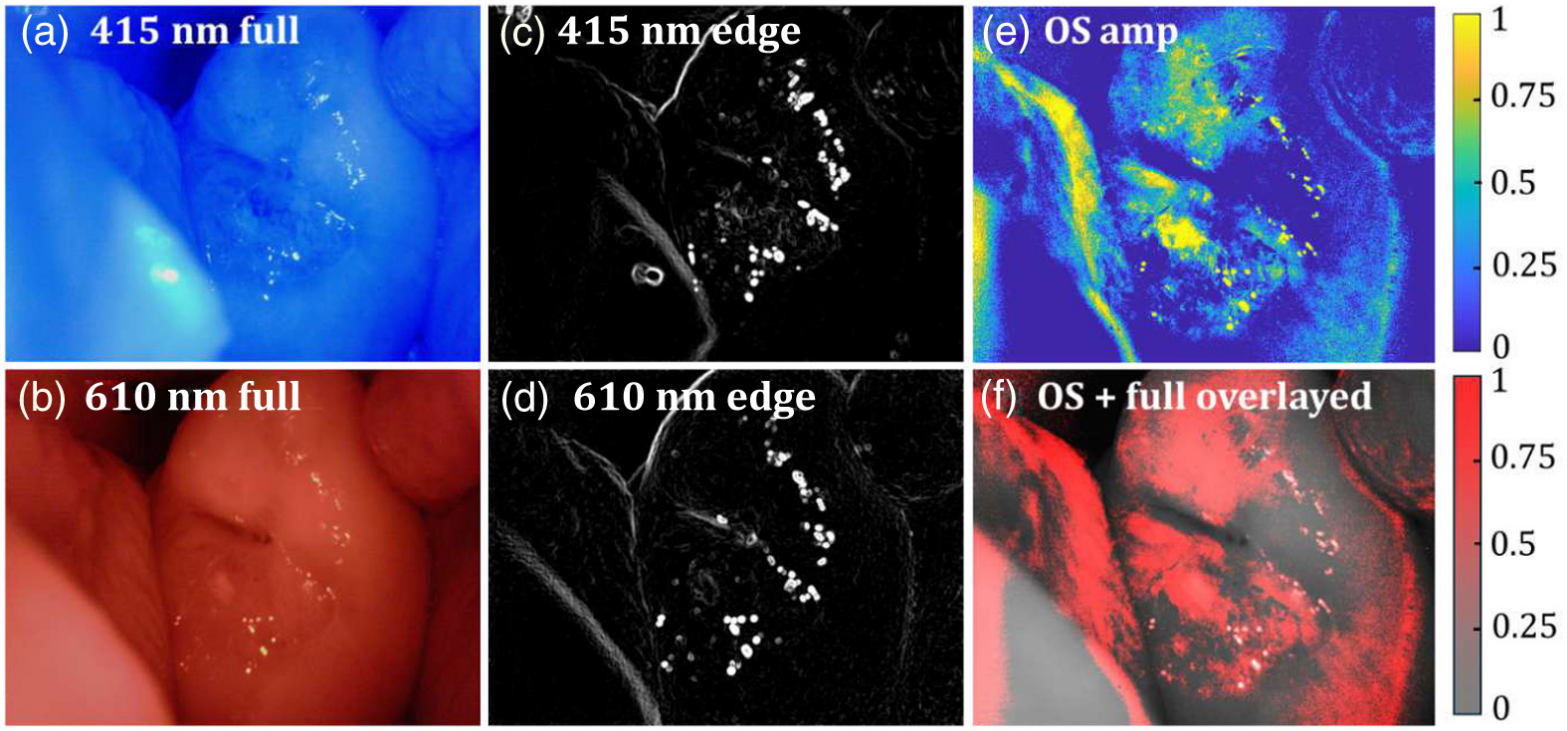
(a), (b) Spectral images at 450 and 610 nm of patient 1. (c), (d) Processed edge images for registration at 450 and 610 nm of patient 1. (e) OS map of patient 1. (f) OS map overlaid on the original full image of patient 1.

The relative OS maps generated from the multispectral images captured at 450 and 610 nm provided a clear visualization of the spatial distribution of tissue oxygenation. The maps effectively highlighted regions with varying oxygenation levels, with hypoxic areas appearing distinctly in blue and oxygen-rich areas in yellow, as per the applied normalized color map, as shown in Fig. 6(e). Figure 6(f) shows the overlaying of the OS maps on the original grayscale cervical images; the anatomical context was preserved, enabling precise localization of oxygenation variations. We used the HSV color space to generate the overlay image as

H←0,  S←1,  V←OS map

In addition, the HSV image is transferred into the RGB color space to obtain the red areas, which emphasize the oxygen-concentrated areas.

The proposed image registration pipeline ensured the accurate alignment of multispectral images, minimizing pixel shifts and artifacts. The analysis revealed a consistent pattern of reduced OS in specific regions, potentially indicative of pathological changes. These results validate the utility of the relative OS mapping approach in providing functional insights into cervical tissue oxygenation. However, the limitation of the two-wavelength oximetry method is that it assumes a negligible scattering effect to simplify the OS estimation of the cervix. This could be one of the limitations of this method because it neglects the scattering effect, especially in biological tissues could impact the accuracy in mapping the OS of the cervix.

### 3.3 Clinical Validation of GynoSight v2.0

The validation of the developed GynoSight v2.0 for six patients with the traditional colposcopy, pap smear, and biopsy is summarized in Table 1. In the case of patient 1, the clinical diagnosis was normal, with acetic acid uptake and iodine negativity noted at the 4 to 5 o’clock position. These findings were consistent with the assessment from GynoSight v2.0. The biopsy report shows stratified squamous epithelium with no dysplasia, confirming the normal diagnosis. The statistical analysis to quantify the shadowing effect shows that the MPI of GynoSight v2.0 images is 1.72-fold higher than colposcopy images, the SAP of colposcopy images is 5.5-fold higher than GynoSight v2.0 images, and the entropy of GynoSight v2.0 images is 1.03-fold higher than colposcopy images. Patient 2 was diagnosed as premalignant with no acetic acid uptake and an iodine-negative region. These findings were consistent with the assessment from GynoSight v2.0. The biopsy report shows morphological and immunohistochemical features consistent with papillary serous carcinoma of ovarian origin, confirming the premalignant diagnosis. Furthermore, the quantitative analysis of the shadowing effect revealed that the MPI of GynoSight v2.0 is 1.65-fold higher than the colposcopy images, the SAP of colposcopy images is 1.27-fold higher than GynoSight v2.0 images, and the entropy of GynoSight v2.0 images is 1.06-fold higher than the colposcopy images. Patient 3 was diagnosed with a high-grade squamous intraepithelial lesion (HSIL) with acetic acid uptake at 7 to 8 o’clock and at 4 to 5 o’clock has lugol’s iodine negative. In addition, these findings were consistent with the assessment from GynoSight v2.0. Quantitatively, the MPI of GynoSight v2.0 images is 1.5-fold higher, SAP is 1.71-fold lower, and entropy is 1.09-fold higher than the colposcopy images, suggesting improved visualization with reduced shadowing. In the case of patient 4, the clinical diagnosis was normal, with acetic acid uptake and iodine negativity noted at the 6 o’clock position. These findings were consistent with the assessment from GynoSight v2.0. The biopsy report shows endocervical tissue with immature squamous metaplasia, confirming the normal diagnosis. GynoSight v2.0 images demonstrated better illumination and reduced shadowing effect. The MPI is found to be 1.2-fold higher, SAP is 7.53-fold lower, and entropy is 1.06-fold higher for GynoSight v2.0 images compared with colposcopy images. Patient 5, diagnosed with HSIL, had dense acetowhite changes and iodine-negative regions, and biopsy confirmed HSIL. In addition, quantitative analysis of the shadowing effect shows that the MPI of GynoSight v2.0 images is 2-fold higher, SAP is 8.20-fold lower, and entropy is 1.02-fold higher than the colposcopy images. Last, patient 6, diagnosed with HSIL, presented with extensive hazy acetowhite areas at 12 o’clock and iodine negative across all quadrants; GynoSight v2.0 accurately identified all iodine-negative regions, with biopsy confirming HSIL. The statistical analysis of the shadowing effect shows that GynoSight v2.0 images demonstrated better illumination and reduced shadowing effect. The MPI is found to be 2-fold higher, SAP is 2.82-fold lower, and entropy is 1.07-fold higher for GynoSight v2.0 images compared with colposcopy images. Across all six cases, GynoSight v2.0 was able to locate acetic acid uptake and iodine-negative regions that closely matched clinical observations and biopsy results. Overall, the results showed good agreement with clinical diagnoses, highlighting the potential of GynoSight as a reliable diagnostic aid with reduced shadowing effect.

**Table 1:** Clinical findings.

SN	Clinical impression	Assessment from GynoSight v1.0
1	**Diagnosis**: normal	• **Acetic acid uptake**: 4 to 5 o’clock and 9 o’clock
	**TZ type**—type 1	• **Lugol’s iodine** **negative**: 4 to 5 o’clock
	**Pap smear report**—NILM	
	**Acetic acid uptake**—hazy acetowhite uptake at 4 to 5 o’clock and dense uptake at 9 o’clock	• **Shadowing effect:** MPI of GynoSight v2.0 was 1.72 times that of colposcopy, SAP of colposcopy is 5.5 times that of GynoSight v2.0, entropy of GynoSight v2.0 is 1.03 times that of colposcopy
	**Lugol’s iodine** **negative**—4 to 5 o’clock	
	**Others**—no abnormal vessels	
	**Biopsy report**—stratified squamous epithelium with no dysplasia/loss of polarity	
2	**Diagnosis**: premalignant	• **Acetic acid uptake**: no acetic acid uptake
	**TZ type**—type 3	
		• **Lugol’s iodine** **negative:** no iodine-negative region seen
	**Pap smear report**—ASC-H	
	**Acetic acid uptake**—no acetowhite area	• **Shadowing effect:** MPI of GynoSight v2.0 was 1.65 times that of colposcopy, SAP of colposcopy is 1.27 times that of GynoSight v2.0, entropy of GynoSight v2.0 is 1.06 times that of colposcopy
	**Lugol’s iodine** **negative**—no iodine negative area	
	**Others**—no abnormal vessels	
	**Biopsy report**—morphological and immunohistochemical features are consistent with papillary serous carcinoma of ovarian origin	
3	**Diagnosis**: malignant	• **Acetic acid uptake**: 7 to 8 o’clock
	**TZ type**—type 2	• **Lugol’s iodine** **negative:** 4 to 5 o’clock
	**Pap smear report**—HSIL	
	**Acetic acid uptake**—7 to 8 o’clock	• **Shadowing effect:** MPI of GynoSight v2.0 was 1.5 times that of colposcopy, SAP of colposcopy is 1.71 times that of GynoSight v2.0, entropy of GynoSight v2.0 is 1.09 times that of colposcopy
	**Lugol’s iodine** **negative**—4 to 5 o’clock	
	**Others**—4 to 5 o’clock and 7 to 8 o’clock	
	**Biopsy report**—high-grade squamous intraepithelial lesion (HSIL)	
4	**Diagnosis**: normal	• **Acetic acid uptake**: 6 o’clock
	**TZ type**—type 2	• **Lugol’s iodine** **negative:** 6 o’clock
	**Pap smear report**—NILM	
	**Acetic acid uptake**—6 o’clock	• **Shadowing effect:** MPI of GynoSight v2.0 was 1.2 times that of colposcopy, SAP of colposcopy is 7.53 times that of GynoSight v2.0, entropy of GynoSight v2.0 is 1.06 times that of colposcopy
	**Lugol’s iodine** **negative**—6 o’clock	
	**Others**—low-grade lesion at 6 o’clock	
	**Biopsy report**—endocervical tissue with immature squamous metaplasia	
5	**Diagnosis**: malignant	• **Acetic acid uptake**: 2 and 9 o’clock
	**TZ type**—type 1	• **Lugol’s iodine** **negative:** 9 o’clock
	**Pap smear report**—NILM	
	**Acetic acid uptake**—dense uptake at 2 and 9 o’clock	• **Shadowing effect:** MPI of GynoSight v2.0 was 2 times that of colposcopy, SAP of colposcopy is 8.20 times that of GynoSight v2.0, entropy of GynoSight v2.0 is 1.02 times that of colposcopy
	**Lugol’s iodine negative**—9 o’clock	
	**Others**—coarse vessel at 2 o’clock	
	**Biopsy report**—high-grade squamous intraepithelial lesion (HSIL)	
6	**Diagnosis**: malignant	• **Acetic acid uptake**: 12 o’clock
	**TZ type**—type 3	• **Lugol’s iodine** **negative:** in all quadrants
	**Pap smear report**—HSIL	
	**Acetic acid uptake**—hazy acetowhite area at 12 o’clock	• **Shadowing effect:** MPI of GynoSight v2.0 was 2 times that of colposcopy, SAP of colposcopy is 2.82 times that of GynoSight v2.0, entropy of GynoSight v2.0 is 1.07 times that of colposcopy
	**Lugol’s iodine negative**—in all quadrants	
	intraepithelial lesion (HSIL)	
	**Biopsy report**—high-grade squamous	

TZ, transformation zone; LSIL, low-grade intraepithelial lesion; NILM, negative for intraepithelial lesion or malignancy; HSIL, high-grade squamous intraepithelial lesion; ASC-H, atypical squamous cells—cannot exclude high-grade squamous intraepithelial lesion.

### 3.4 Clinical Comparison of GynoSight v2.0 with Biopsy and Colposcope

**Fig. 7:**
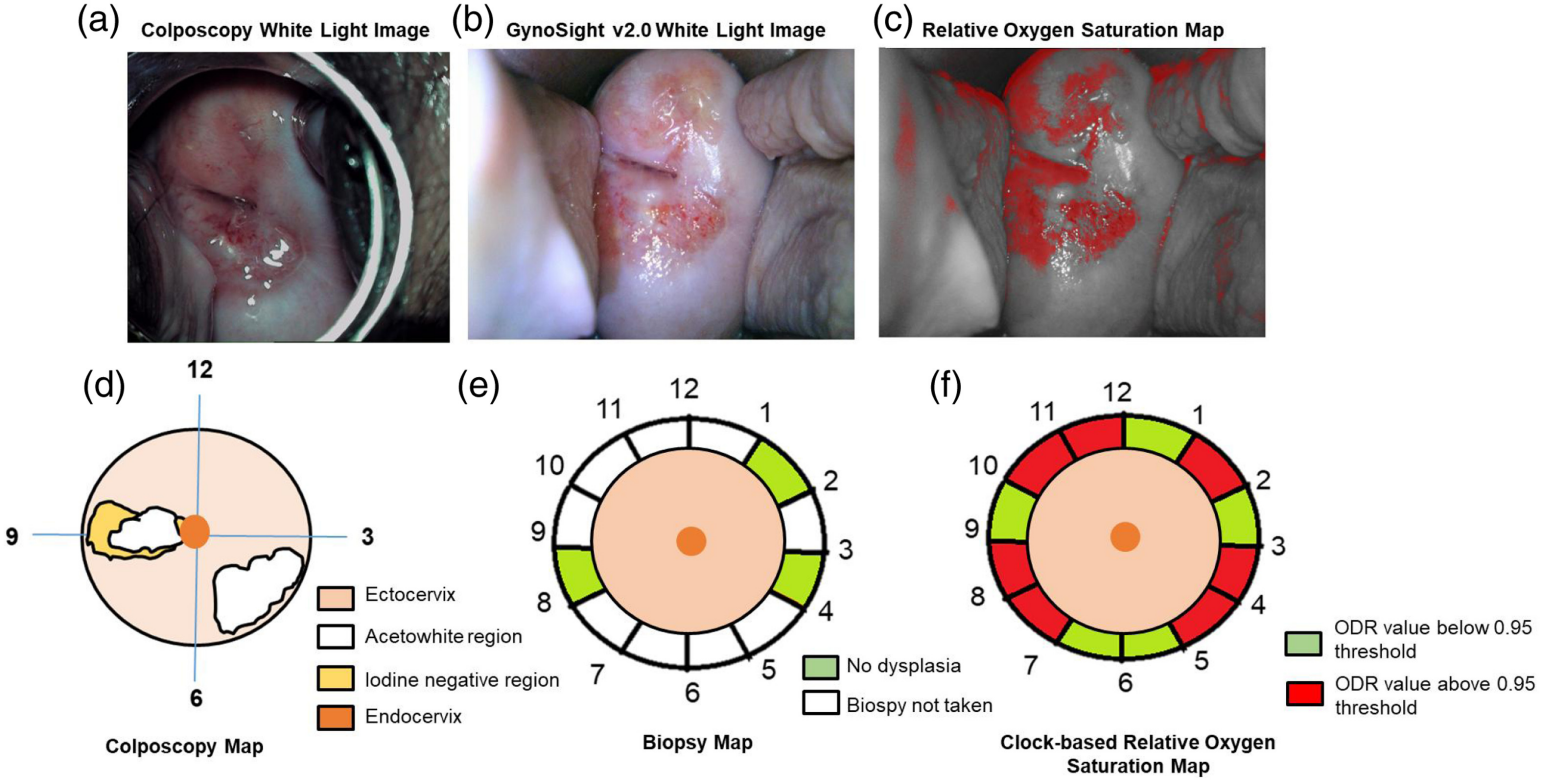
Patient 1: normal subject. (a) Colposcopy white light imaging under application of normal saline. (b) GynoSight v2.0 white light imaging under application of normal saline. (c) Relative OS map after applying a threshold of 0.95. (d) Colposcopy map. (e) Biopsy map. (f) OS map.

**Fig. 8:**
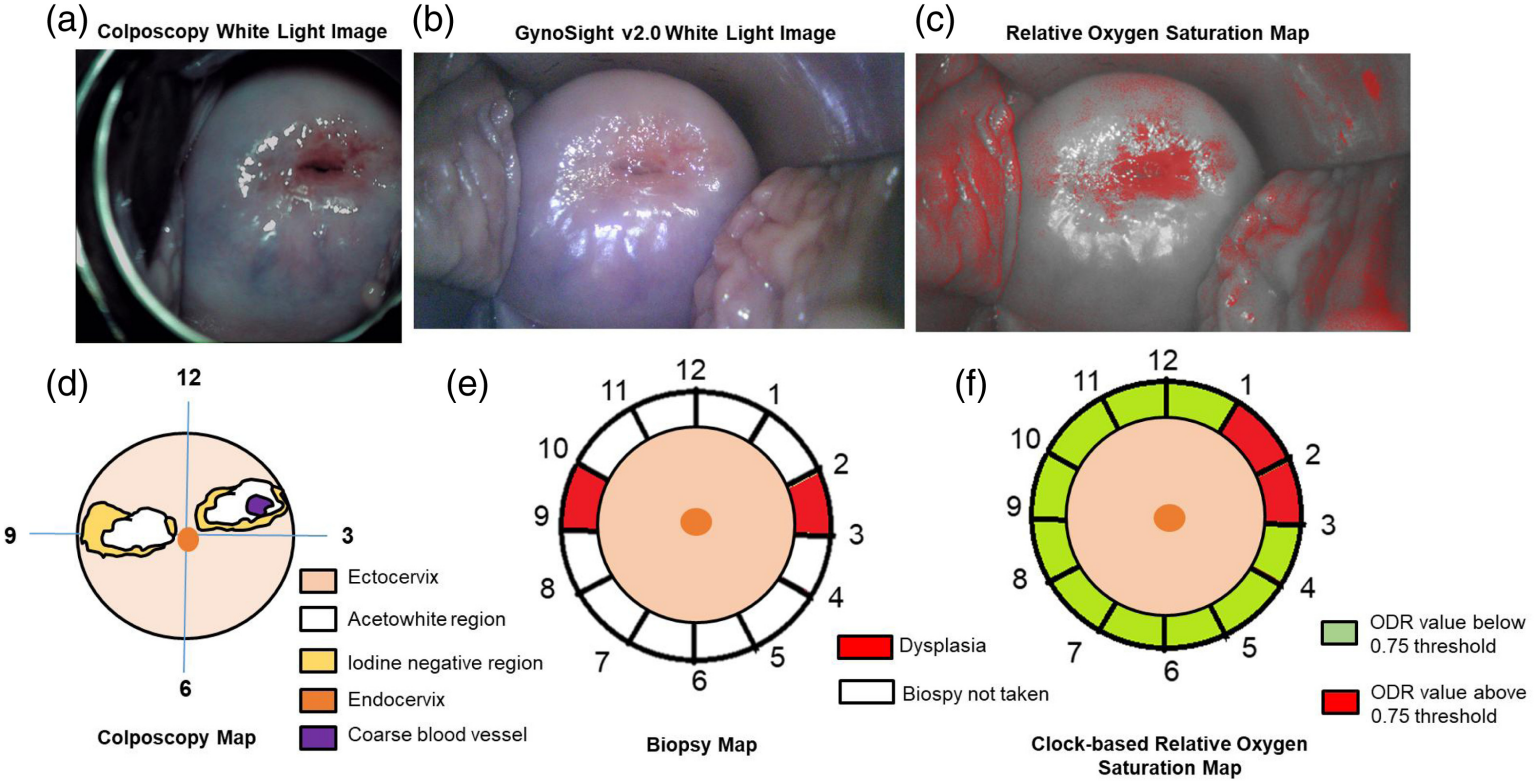
Patient 5: malignant subject. (a) Colposcopy white light imaging under application of normal saline. (b) GynoSight v2.0 white light imaging under application of normal saline. (c) Relative OS map after applying a threshold of 0.75. (d) Colposcopy map. (e) Biopsy map. (f) Relative OS map.

Moreover, patient 1, clinically diagnosed as normal, underwent imaging with both colposcopy and GynoSight v2.0, which is shown in Figs. 7(a) and 7(b). The colposcopy map in Fig. 7(d) shows that the acetowhite region at 4 to 5 and 9 o’clock positions and the iodine negative region at the 9 o’clock position overlap with the acetowhite region. Furthermore, the biopsy taken from 1, 3, and 8 o’clock positions confirms no dysplasia, as shown in Fig. 7(e). The clock-based relative OS map, as shown in Fig. 7(f), showed ODR values above the 0.95 threshold in red (1, 3, 4, 7, 8, 10, and 11 o’clock positions), indicating elevated ODR values. However, these elevated ODR values are due to the presence of superficial blood vessels in the endocervix region, which can influence local reflectance independent of dysplastic changes in the cervix. In contrast to the findings of patient 1, patient 5 is clinically diagnosed as cervical intraepithelial neoplasia grade 3 (CIN3). The colposcopy image and its corresponding colposcopy map are shown in Figs. 8(a) and 8(d), respectively. The colposcopic findings indicate acetowhite and iodine negative in 2 and 9 o’clock positions and a coarse blood vessel pattern in the 2 o’clock position. The biopsy map, as shown in Fig. 8(e), also confirms dysplastic cells in 2 and 9 o’clock positions. The relative OS map closely aligns with the biopsy map, i.e., the clock-based relative OS map in Fig. 8(f) shows an elevated ODR value (above 0.75 ODR threshold) in 1 and 2 o’clock positions.

## 4 Conclusion

The design and development of the non-chip-on-tip-based portable and handheld multispectral diffuse optical transvaginal imaging probe (GynoSight v2.0) were discussed in this work. The transvaginal imaging probe could provide insights into acetic acid and Lugol’s Iodine uptake, blood vessel patterns, and OS levels, enabling early detection of cervical abnormalities. The comparative statistical analysis (MPI, entropy, and SAP) of images from traditional colposcopy and GynoSight v2.0 showcased reduced shadowing effects, enhancing the diagnostic accuracy. The GynoSight v2.0 has the potential for cervical screening as a point-of-care diagnostic and facilitating timely interventions.

Future work will focus on enhancing the quantitative accuracy of mapping the OS of the cervix by considering the scattering effect. In addition, validating the OS map using optical phantoms with known oxygenation values needs to be performed.

## Supplementary Material

10.1117/1.JBO.30.10.106002.s01

## Data Availability

The data that support the findings of this article are not publicly available due to ethical concerns. They can be requested from the author at uttampal@iiitdm.ac.in.
