# Supplementary material for: Development and clinical validation of a clinically translatable non-chip-on-tip transvaginal imaging system (GynoSight v2.0) for early detection of premalignant cervical lesions
Source: J Biomed Opt. 2025 Oct 27;30(10):106002. doi: 10.1117/1.JBO.30.10.106002 (PMC12558412; doi:10.1117/1.JBO.30.10.106002)
Supplement: Supplementary file 1 [file JBO_030_106002_SD001.pdf]

Development and Clinical Validation of a Clinically Translatable Non-Chip-on-Tip Transvaginal Imaging System (GynoSight v2.0) for Early Detection of Premalignant Cervical Lesions

Karthika J,[1] Mohammed Ansar PT,[2] Keerthana A,[3] Milind Lal,[4] Dhanush Koodi,[5] Arpitha Anantharaju,[6] Rongguang Liang,[7] YuanYuan Sun,[8] Uttam M.Pal[9#]

[1] Department of Sciences and Humanities, Indian Institute of Information Technology, Design and Manufacturing, Kancheepuram, Chennai, 600127, Tamil Nadu, India.
[2] Department of Electronics and Communications, Indian Institute of Information Technology, Design and Manufacturing, Kancheepuram, Chennai, 600127, Tamil Nadu, India.
3 Department of Gynecology and Obstetrics, Jawaharlal Institute of Postgraduate Medical Education & Research, Puducherry, 605006, Puducherry, India.
[4] Department of Mechanical Engineering, National Institute of Engineering, Mysore, 570008, Karnataka, India.
[5] Department of Gynecology and Obstetrics, Jawaharlal Institute of Postgraduate Medical Education & Research, Puducherry, 605006, Puducherry, India.
[6] Department of Electronics and Communication Engineering, Sri Sairam Engineering College Chennai, 602109, Tamil Nadu, India.
[7] Wyant College of Optical Sciences at The University of Arizona, Tucson, 85721, Arizona, United States.
[8] Wyant College of Optical Sciences at The University of Arizona, Tucson, 85721, Arizona, United States.
[9] Department of Electronics and Communications, Indian Institute of Information Technology, Design and Manufacturing, Kancheepuram, Chennai, 600127, Tamil Nadu, India.
[#] correspondence e-mail: uttampal@iiitdm.ac.in

S1. Graphical User Interface

The system's graphical user interface (GUI) is designed for ease of use and consists of four key panels, as shown in Fig.S1. The patient detail entry panel allows clinicians to input patient information such as name, age, and patient ID. The test mode selection panel enables the user to choose from various diagnostic modes like normal saline, VIA (visual inspection with acetic acid), and VILI (visual inspection with lugol's iodine), ensuring flexibility in testing. The Image Acquisition panel displays real-time imaging in two modes, one in normal mode and another in AI-assisted mode, to detect atypical blood vessels, acetowhite, and iodine-negative areas in the cervix, where users can capture cervical images using the integrated camera. Finally, the data analysis panel presents raw, processed, and AI-annotated images to aid in the interpretation and diagnosis of cervical abnormalities.

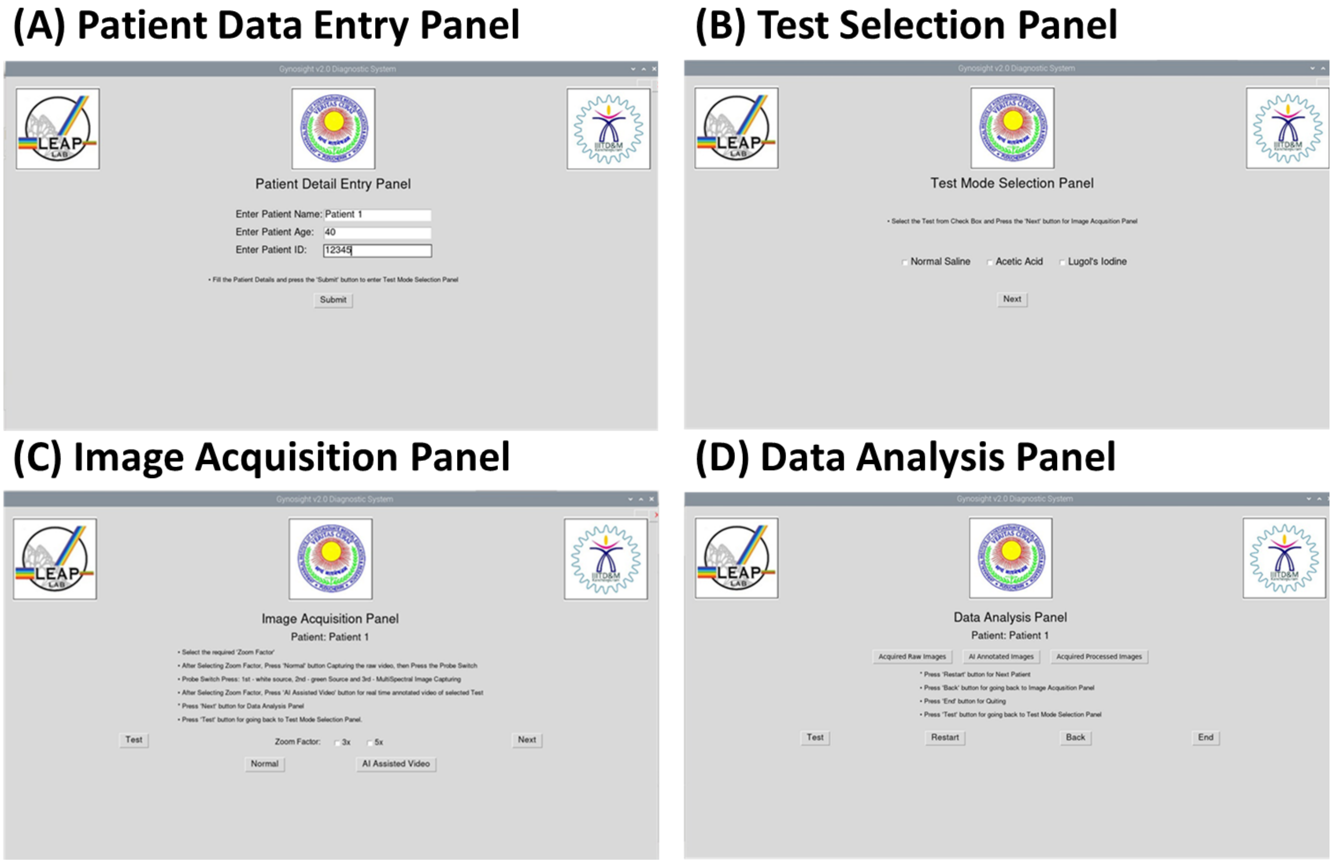

Fig S1: GUI for GynoSight v2.0. (A) Patient details entry panel. (B) Test mode (normal saline, acetic acid, & lugol's iodine) selection panel. (C) Image acquisition panel. (D) Data analysis panel to view the acquired images.

S2. GynoSight v2.0 Imaging Protocol

The protocol for acquiring images using GynoSight v2.0 for clinical evaluation is as follows: (1) Connect the probe and the monitor to the electronic box. (2) Calibrate the system to optimize settings. (3) Obtain and document patient consent before the procedure. (4) Access the system interface and navigate to the patient information section to enter the patient details. (5) Verify that the patient is not currently menstruating and position the patient comfortably in the examination area (e.g., in lithotomy position), ensuring privacy and adequate support. (6) Gently insert a speculum to open the vaginal canal for clear access and apply normal saline using a cotton swab. (7) Position the probe at a working distance of 4cm away from the surface of the cervix and keep the probe steady to avoid motion artifacts. (8) Press the push button, click 1 to turn two pairs of white LEDs and identify the ROI (Region of Interest), click 2 to turn two pairs of green LEDs to identify the presence of abnormal blood vessels, and click 3 to turn multispectral light source one after the other with the time interval of 5 secs and the images are stored simultaneously. The system's timing diagram is shown in Fig.1(F). (9) Review images for quality check and retake if necessary. (10) Repeat procedures (8) and (9) for capturing images after applying acetic acid and lugol's iodine. (11) Sterilize the probe sleeve using ethanol. The schematic of the image acquisition protocol is given in Fig.S2.

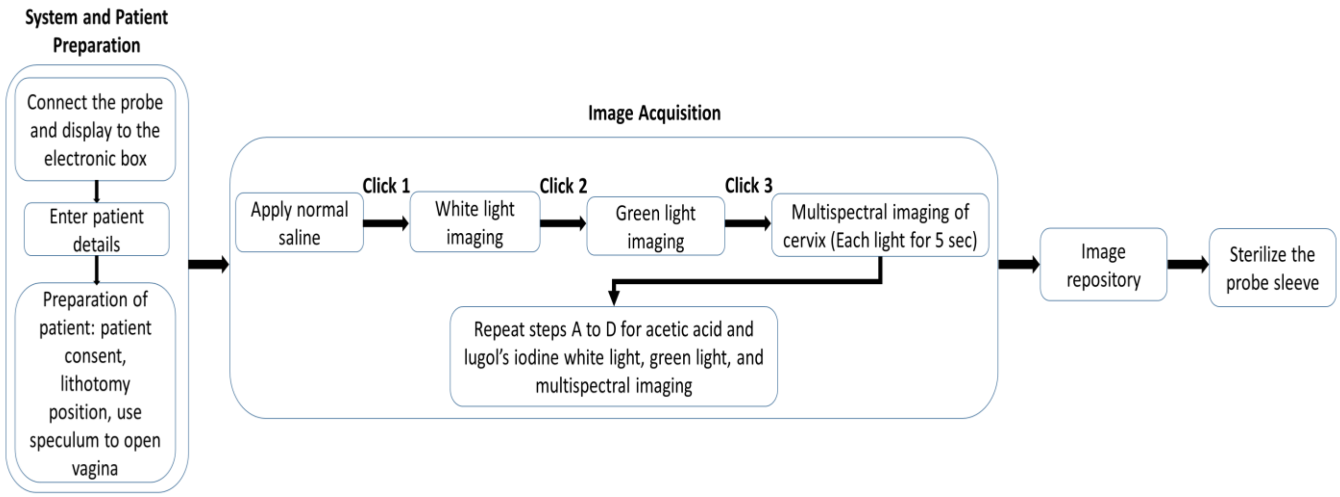

Fig.S2: Schematic representation of image acquisition protocol. The first step involves system and patient preparation. The next step is the image acquisition by illuminating the tissues using white light, green light, and then multispectral. The final step is image storage.

TABLE SII
IMAGING QUALITY ASSESSMENT PARAMETERS

Patient No.	Mean Pixel Intensity (MPI)		Standard Deviation		Entropy		Contrast to Noise Ratio (CNR)		Shadow Area Percentage (SAP)	
	Colposcopy	GynoSight v2.0	Colposcopy	GynoSight v2.0	Colposcopy	GynoSight v2.0	Colposcopy	GynoSight v2.0	Colposcopy	GynoSight v2.0
1	78.28	134.95	34.85	39.14	6.97	7.23	3.42	2.73	48.75	8.73
2	66.16	109.64	51.23	41.84	6.90	7.37	3.38	3.02	47.39	37.13
3	82.88	129.14	24.74	41.51	6.56	7.20	2.68	3.44	17.76	10.37
4	80.40	98.32	41.33	26.14	7.10	6.66	2.76	3.17	31.19	4.14
5	63.66	127.62	45.72	34.81	6.79	6.98	5.17	3.24	41.52	5.06
6	46.10	94.83	40.66	37.44	6.65	7.14	4.46	3.55	54.29	19.24

TABLE SI

TECHNICAL SPECIFICATIONS

Components	Specifications
Camera	Y001
LEDs	White (SKU: 827905), 450 nm (SKU: 827900), 545 nm (SKU:827901), 620 nm (SKU: 827902)
Display	10.1 inch waveshare capacitive touch display
Power supply	Starwatt Mini UPS for 12V Router and 5V
Working distance of probe	4 cm
Camera sensor	GC5035 imaging sensor
Dimensions of electronic box	Length = 225mm, Width = 225mm, Height = 35mm
Dimensions of Probe:<br>1. Probe length<br>2. Distal end diameter	 239.30 mm<br>19.50 mm
Image acquisition time (per LED)	5 sec
Light illumination interval (per LED)	5 sec
One complete image acquisition cycle	25 sec
Processor	Raspberry Pi 5 Model 8GB
Cost	INR 90,000
